# Supplementary material for: A mixture of experts (MoE) model to improve AI-based computational pathology prediction performance under variable levels of image blur
Source: BMC Med Imaging. 2025 Oct 13;25:407. doi: 10.1186/s12880-025-01974-w (PMC12516837; doi:10.1186/s12880-025-01974-w)
Supplement: Supplementary file 1 — Supplementary Material 1 [file 12880_2025_1974_MOESM1_ESM.pdf]

Supplementary materials

A Mixture of Experts (MoE) model to improve AI-based computational pathology prediction performance under variable levels of histopathology image blur

Table S1. Data split at each CV for grading task

	CV Folds	Division of Training Sets into 5 Training and 5 Tuning Sets for Each Cross-Validation Fold
CV1	Training set 1 (N = 741)<br>Grade 1 N=293<br>Grade 3 N=448<br><br>Validation set1 (N = 175)<br>Grade 1 N = 70<br>Grade 3 N = 105	Training set 1 (N = 592)<br>Grade 1 N = 234<br>Grade 3 N = 358<br>Tuning set 1 (N = 149)<br>Grade 1 N = 59<br>Grade 3 N = 90
CV2	Training set 1 (N = 742)<br>Grade 1 N=295<br>Grade 3 N=447<br><br>Validation set1 (N = 174)<br>Grade 1 N = 68<br>Grade 3 N = 106	Training set 1 (N = 593)<br>Grade 1 N = 236<br>Grade 3 N = 357<br>Tuning set 1 (N = 149)<br>Grade 1 N = 59<br>Grade 3 N = 90
CV3	Training set 1 (N = 748)<br>Grade 1 N=296<br>Grade 3 N=452<br><br>Validation set1 (N = 168)<br>Grade 1 N = 67<br>Grade 3 N = 101	Training set 1 (N = 598)<br>Grade 1 N = 237<br>Grade 3 N = 361<br>Tuning set 1 (N = 150)<br>Grade 1 N = 59<br>Grade 3 N = 91
CV4	Training set 1 (N = 728)<br>Grade 1 N=285<br>Grade 3 N=443<br><br>Validation set1 (N = 188)<br>Grade 1 N = 78<br>Grade 3 N = 110	Training set 1 (N = 582)<br>Grade 1 N = 228<br>Grade 3 N = 354<br>Tuning set 1 (N = 146)<br>Grade 1 N = 57<br>Grade 3 N = 89
CV5	Training set 1 (N = 705)<br>Grade 1 N=283<br>Grade 3 N=422<br><br>Validation set1 (N = 211)<br>Grade 1 N = 80<br>Grade 3 N = 131	Training set 1 (N = 564)<br>Grade 1 N = 226<br>Grade 3 N = 338<br>Tuning set 1 (N = 141)<br>Grade 1 N = 57<br>Grade 3 N = 84

Table S2. Data split at each CV for ER task

	CV Folds	Division of Training Sets into 5 Training and 5 Tuning Sets for Each Cross-Validation Fold
CV1	Training set 1 (N = 1324)<br>ER- N=138<br>ER+ N=1186<br><br>Validation set1 (N = 332)<br>ER- N = 35<br>ER+ N = 297	Training set 1 (N = 1059)<br>ER- N = 110<br>ER+ N = 949<br>Tuning set 1 (N = 265)<br>ER- N = 28<br>ER+ N = 237
CV2	Training set 1 (N = 1325)<br>ER- N=139<br>ER+ N=1186<br><br>Validation set1 (N = 331)<br>ER- N = 34<br>ER+ N = 297	Training set 1 (N = 1060)<br>ER- N = 111<br>ER+ N = 949<br>Tuning set 1 (N = 265)<br>ER- N = 28<br>ER+ N = 237
CV3	Training set 1 (N = 1325)<br>ER- N=139<br>ER+ N=1186<br><br>Validation set1 (N = 331)<br>ER- N = 34<br>ER+ N = 297	Training set 1 (N = 1060)<br>ER- N = 111<br>ER+ N = 949<br>Tuning set 1 (N = 265)<br>ER- N = 28<br>ER+ N = 237
CV4	Training set 1 (N = 1325)<br>ER- N=138<br>ER+ N=1187<br><br>Validation set1 (N = 331)<br>ER- N = 35<br>ER+ N = 296	Training set 1 (N = 1060)<br>ER- N = 110<br>ER+ N = 950<br>Tuning set 1 (N = 265)<br>ER- N = 28<br>ER+ N = 237
CV5	Training set 1 (N = 1325)<br>ER- N=138<br>ER+ N=1187<br><br>Validation set1 (N = 331)<br>ER- N = 35<br>ER+ N = 296	Training set 1 (N = 1060)<br>ER- N = 110<br>ER+ N = 950<br>Tuning set 1 (N = 265)<br>ER- N = 28<br>ER+ N = 237

Table S3. Data split at each CV for PR task

	CV Folds	Division of Training Sets into 5 Training and 5 Tuning Sets for Each Cross-Validation Fold
CV1	Training set 1 (N = 1313)<br>PR- N=377<br>PR+ N=936<br><br>Validation set1 (N = 329)<br>PR- N = 95<br>PR+ N = 234	Training set 1 (N = 1050)<br>PR- N = 301<br>PR+ N = 749<br>Tuning set 1 (N = 263)<br>PR- N = 76<br>PR+ N = 187
CV2	Training set 1 (N = 1314)<br>PR- N=378<br>PR+ N=936<br><br>Validation set1 (N = 328)<br>PR- N = 94<br>PR+ N = 234	Training set 1 (N = 1051)<br>PR- N = 302<br>PR+ N = 749<br>Tuning set 1 (N = 263)<br>PR- N = 76<br>PR+ N = 187
CV3	Training set 1 (N = 1314)<br>PR- N=378<br>PR+ N=936<br><br>Validation set1 (N = 328)<br>PR- N = 94<br>PR+ N = 234	Training set 1 (N = 1051)<br>PR- N = 302<br>PR+ N = 749<br>Tuning set 1 (N = 263)<br>PR- N = 76<br>PR+ N = 187
CV4	Training set 1 (N = 1314)<br>PR- N=378<br>PR+ N=936<br><br>Validation set1 (N = 328)<br>PR- N = 94<br>PR+ N = 234	Training set 1 (N = 1051)<br>PR- N = 302<br>PR+ N = 749<br>Tuning set 1 (N = 263)<br>PR- N = 76<br>PR+ N = 187
CV5	Training set 1 (N = 1314)<br>PR- N=378<br>PR+ N=936<br><br>Validation set1 (N = 328)<br>PR- N = 94<br>PR+ N = 234	Training set 1 (N = 1051)<br>PR- N = 302<br>PR+ N = 749<br>Tuning set 1 (N = 263)<br>PR- N = 76<br>PR+ N = 187

Table S4. Data split at each CV for Her2 task

	CV Folds	Division of Training Sets into 5 Training and 5 Tuning Sets for Each Cross-Validation Fold
CV1	Training set 1 (N = 1271)<br>Her2- N=1130<br>Her2+ N=141<br><br>Validation set1 (N = 318)<br>Her2- N = 283<br>Her2+ N = 35	Training set 1 (N = 1016)<br>Her2- N = 903<br>Her2+ N = 113<br>Tuning set 1 (N = 255)<br>Her2- N = 227<br>Her2+ N = 28
CV2	Training set 1 (N = 1271)<br>Her2- N=1130<br>Her2+ N=141<br><br>Validation set1 (N = 318)<br>Her2- N = 283<br>Her2+ N = 35	Training set 1 (N = 1016)<br>Her2- N = 903<br>Her2+ N = 113<br>Tuning set 1 (N = 255)<br>Her2- N = 227<br>Her2+ N = 28
CV3	Training set 1 (N = 1271)<br>Her2- N=1130<br>Her2+ N=141<br><br>Validation set1 (N = 318)<br>Her2- N = 283<br>Her2+ N = 35	Training set 1 (N = 1016)<br>Her2- N = 903<br>Her2+ N = 113<br>Tuning set 1 (N = 255)<br>Her2- N = 227<br>Her2+ N = 28
CV4	Training set 1 (N = 1271)<br>Her2- N=1131<br>Her2+ N=140<br><br>Validation set1 (N = 318)<br>Her2- N = 283<br>Her2+ N = 35	Training set 1 (N = 1016)<br>Her2- N = 904<br>Her2+ N = 112<br>Tuning set 1 (N = 255)<br>Her2- N = 227<br>Her2+ N = 28
CV5	Training set 1 (N = 1272)<br>Her2- N=1131<br>Her2+ N=141<br><br>Validation set1 (N = 317)<br>Her2- N = 282<br>Her2+ N = 35	Training set 1 (N = 1017)<br>Her2- N = 904<br>Her2+ N = 113<br>Tuning set 1 (N = 255)<br>Her2- N = 227<br>Her2+ N = 28

Table S5. The Median LV values of 10,000 tiles at each sigma value

Sigma Values	Median LV Values
0	2520.674
0.5	1254.291
1.0	199.213
1.5	52.652
2.0	19.608
2.5	9.341
3.0	5.745
3.5	4.172
4.0	3.346
5.0	2.650
6.0	2.389
7.0	2.186
8.0	2.067
9.0	1.965
10.0	1.893

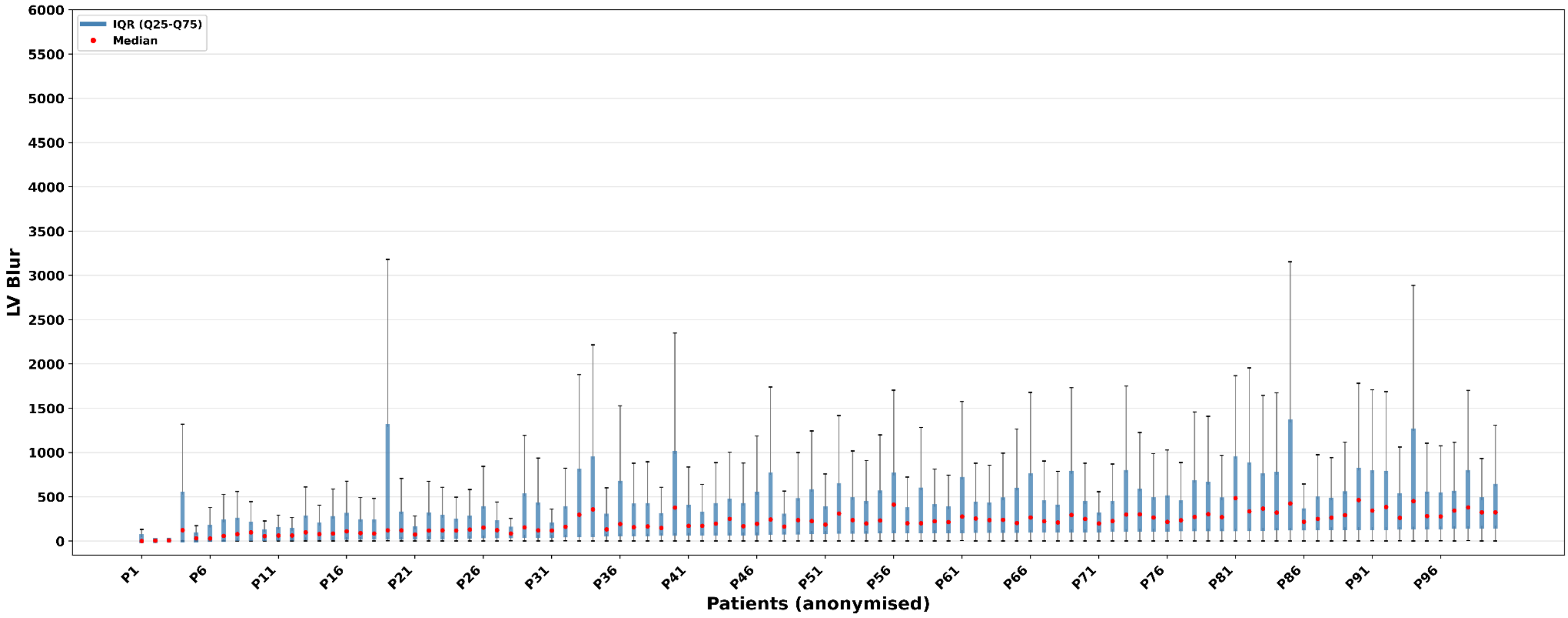

Figure S1. Tile-level LV blur distribution across the first 100 patients (NHG 1 vs. 3) with the highest level of blur in a real-world WSI dataset. For each patient, the blue vertical bars represent the interquartile range (25th–75th percentile) of tile-level LV blur values, the red dots indicate the median LV blur.

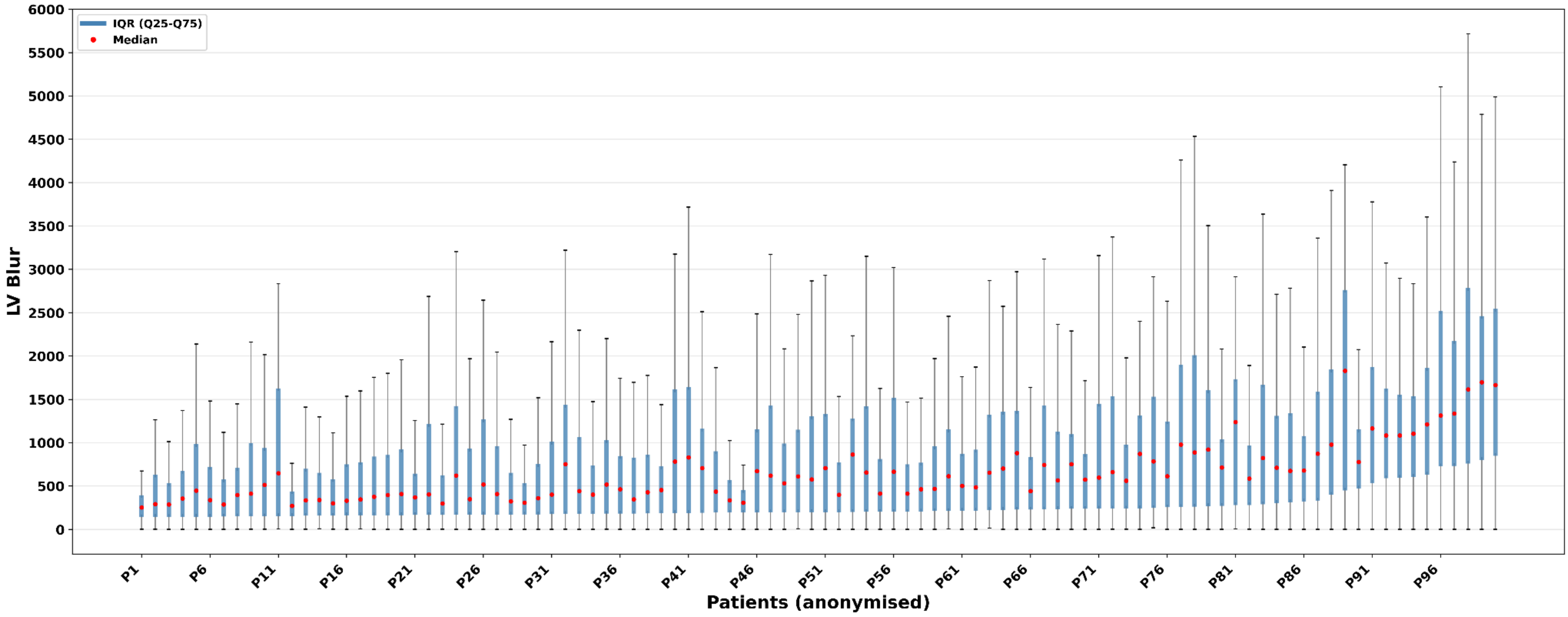

Figure S2. Tile-level LV blur distribution across the last 100 patients (NHG 1 vs. 3) with the highest level of blur in a real-world WSI dataset. For each patient, the blue vertical bars represent the interquartile range (25th–75th percentile) of tile-level LV blur values, the red dots indicate the median LV blur.

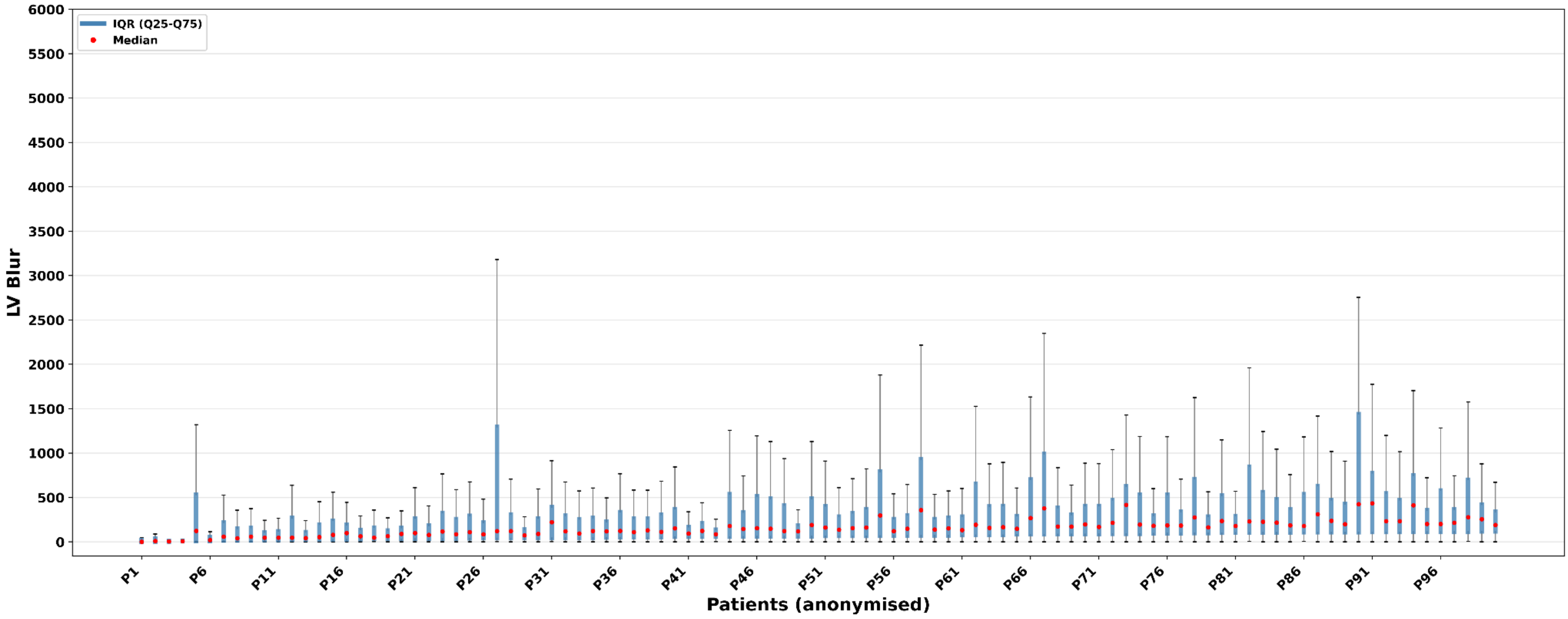

Figure S3. Tile-level LV blur distribution across the first 100 patients (ER) with the highest level of blur in a real-world WSI dataset. For each patient, the blue vertical bars represent the interquartile range (25th–75th percentile) of tile-level LV blur values, the red dots indicate the median LV blur.

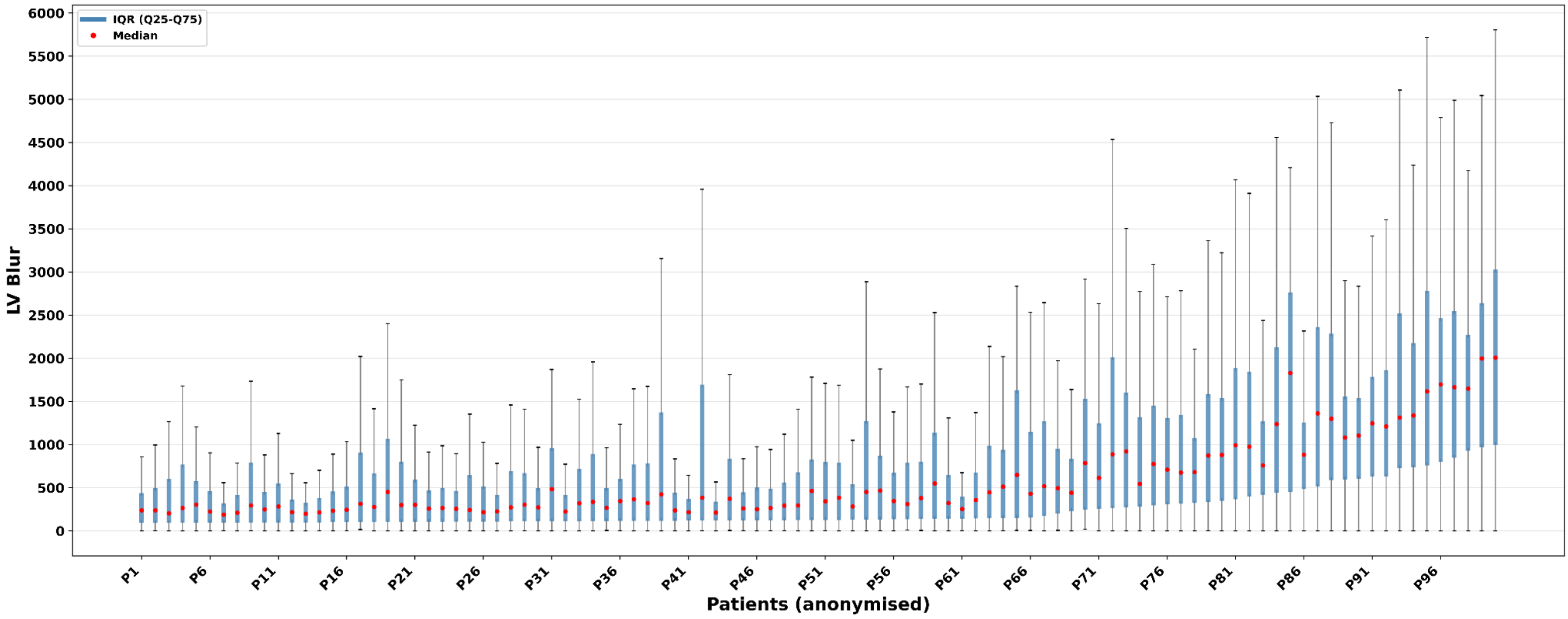

Figure S4. Tile-level LV blur distribution across the last 100 patients (ER) with the highest level of blur in a real-world WSI dataset. For each patient, the blue vertical bars represent the interquartile range (25th–75th percentile) of tile-level LV blur values, the red dots indicate the median LV blur.

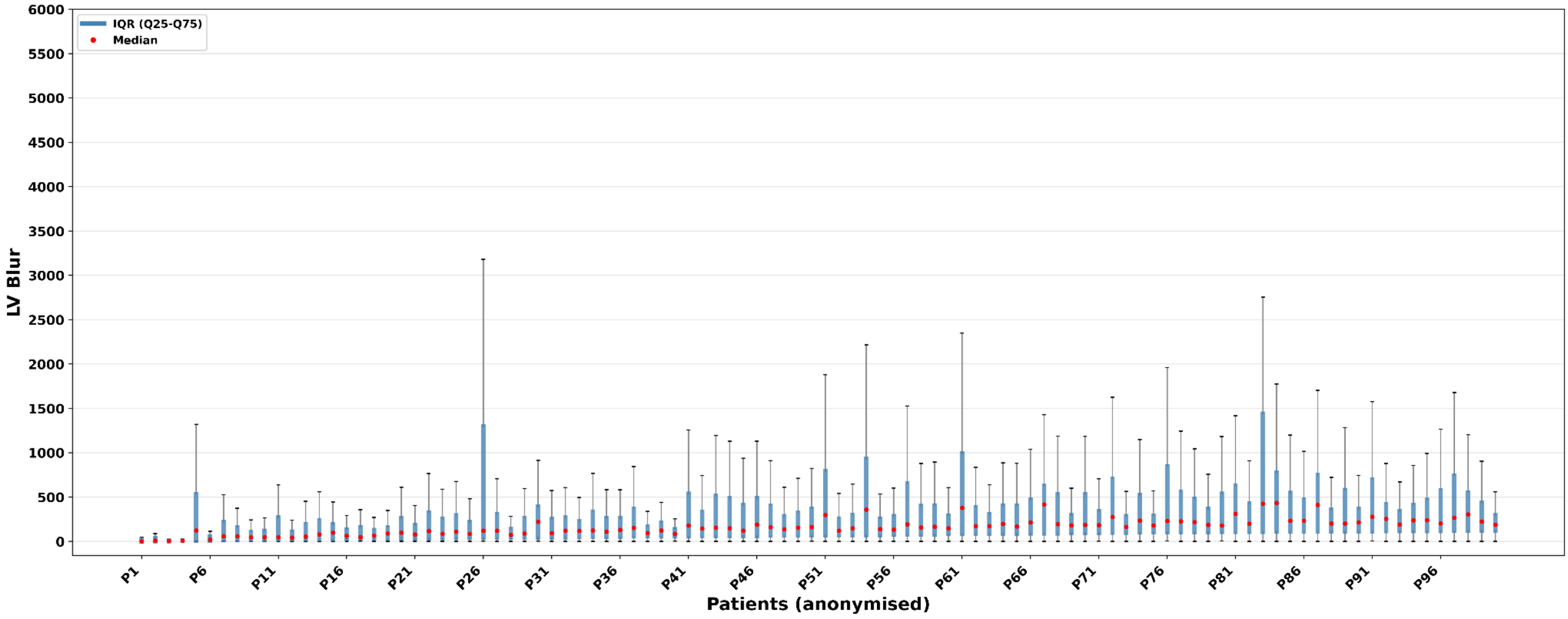

Figure S5. Tile-level LV blur distribution across the first 100 patients (Her2) with the highest level of blur in a real-world WSI dataset. For each patient, the blue vertical bars represent the interquartile range (25th–75th percentile) of tile-level LV blur values, the red dots indicate the median LV blur.

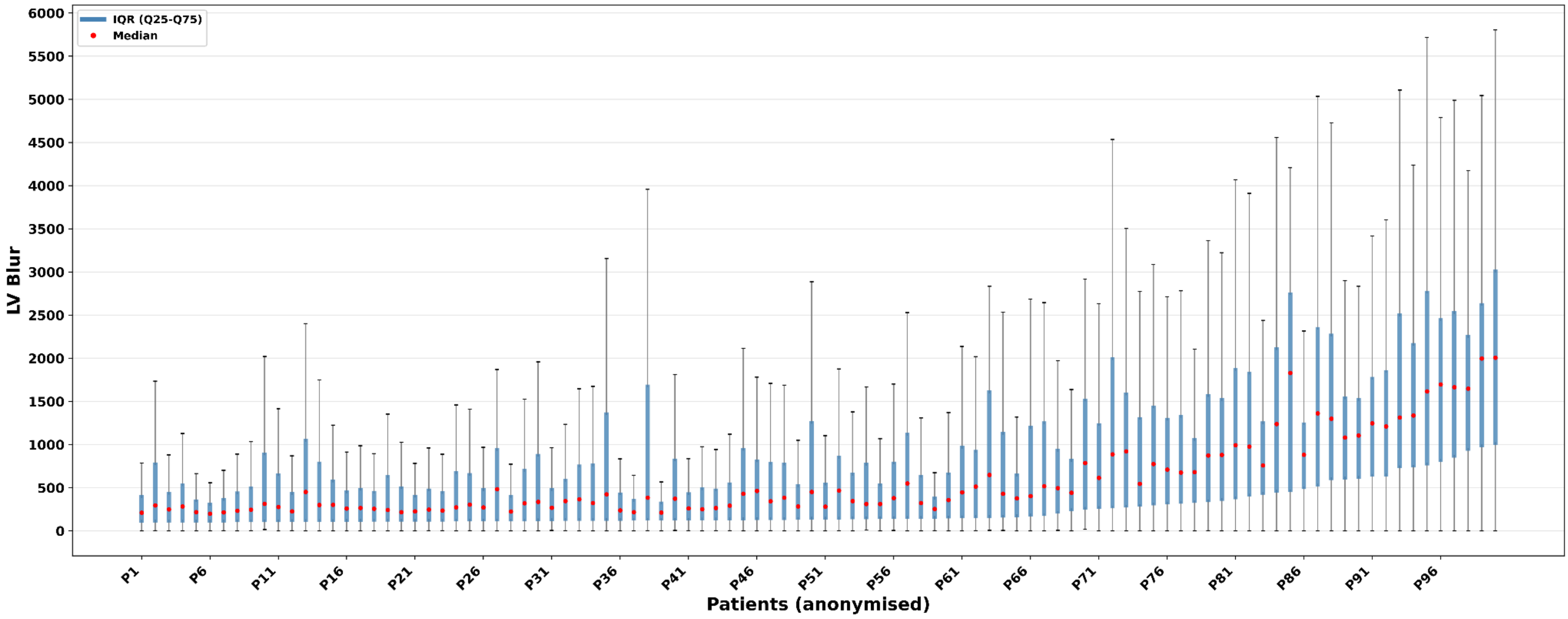

Figure S6. Tile-level LV blur distribution across the last 100 patients (Her2) with the highest level of blur in a real-world WSI dataset. For each patient, the blue vertical bars represent the interquartile range (25th–75th percentile) of tile-level LV blur values, the red dots indicate the median LV blur.

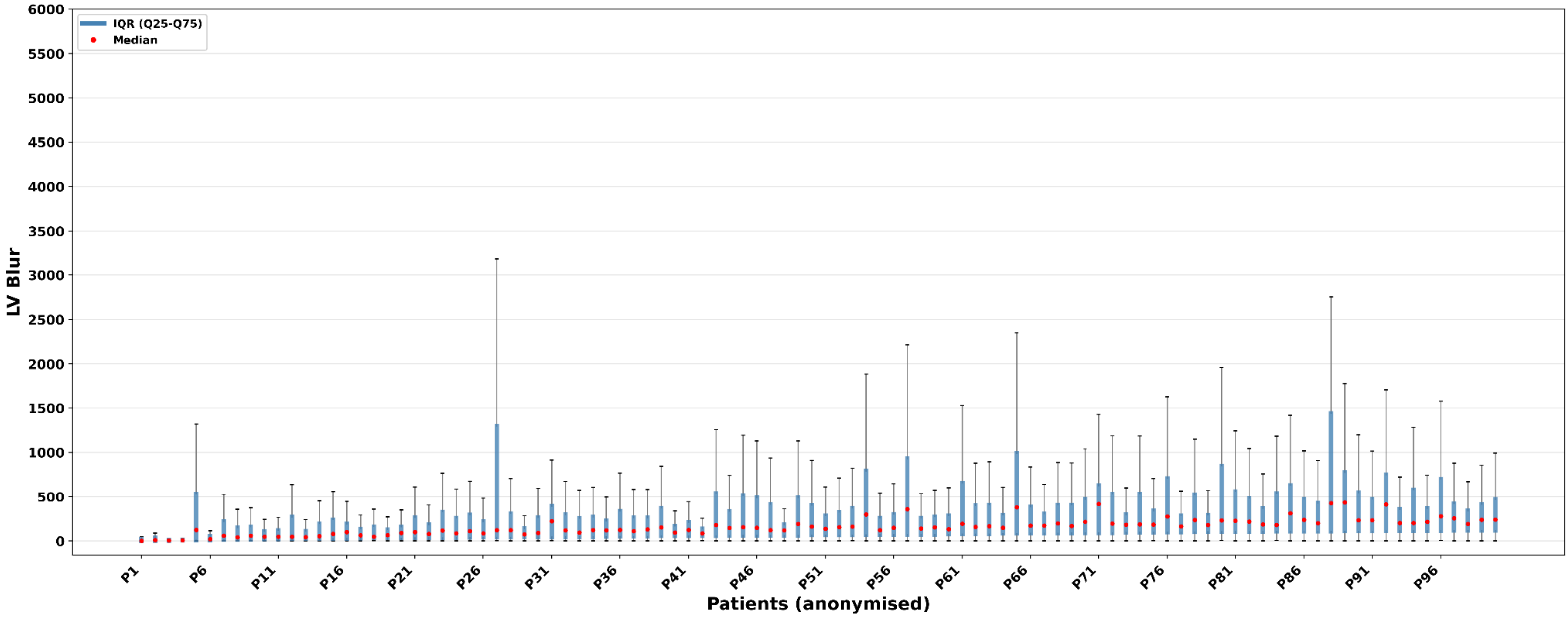

Figure S7. Tile-level LV blur distribution across the first 100 patients (PR) with the highest level of blur in a real-world WSI dataset. For each patient, the blue vertical bars represent the interquartile range (25th–75th percentile) of tile-level LV blur values, the red dots indicate the median LV blur.

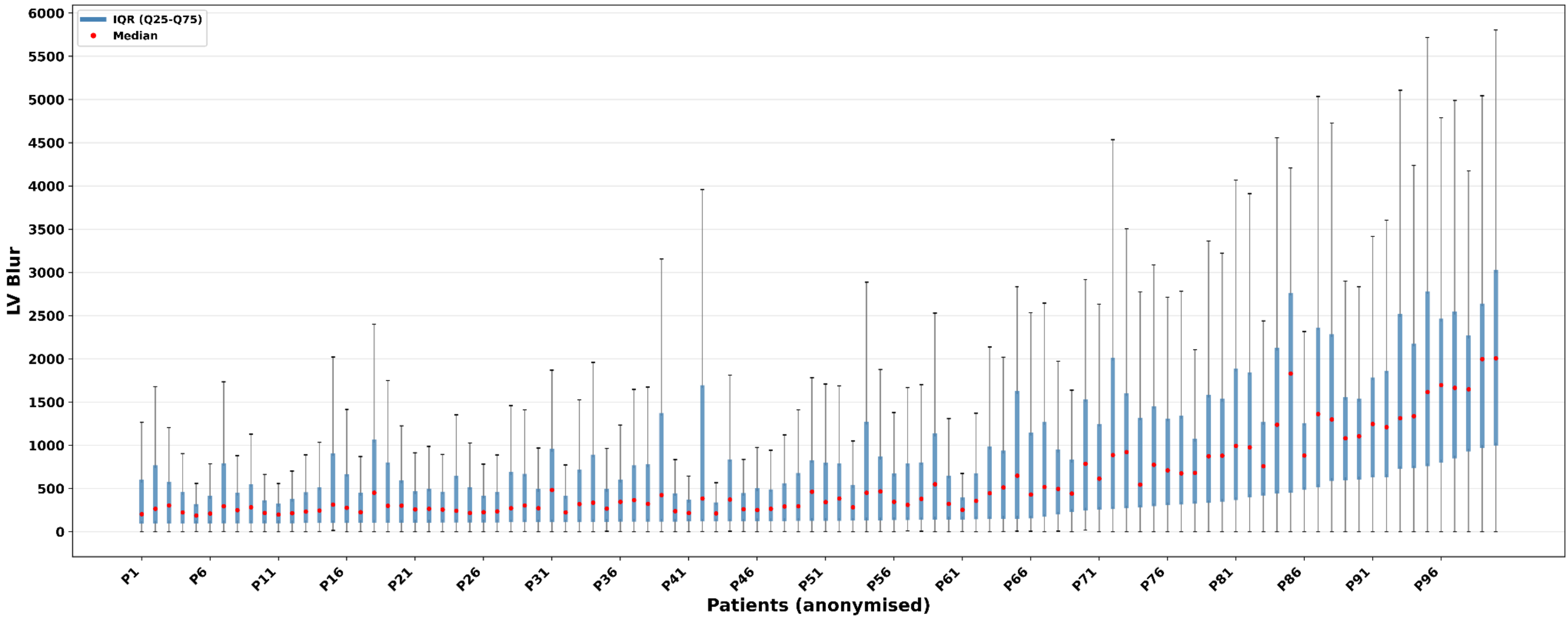

Figure S8. Tile-level LV blur distribution across the last 100 patients (PR) with the highest level of blur in a real-world WSI dataset. For each patient, the blue vertical bars represent the interquartile range (25th–75th percentile) of tile-level LV blur values, the red dots indicate the median LV blur.

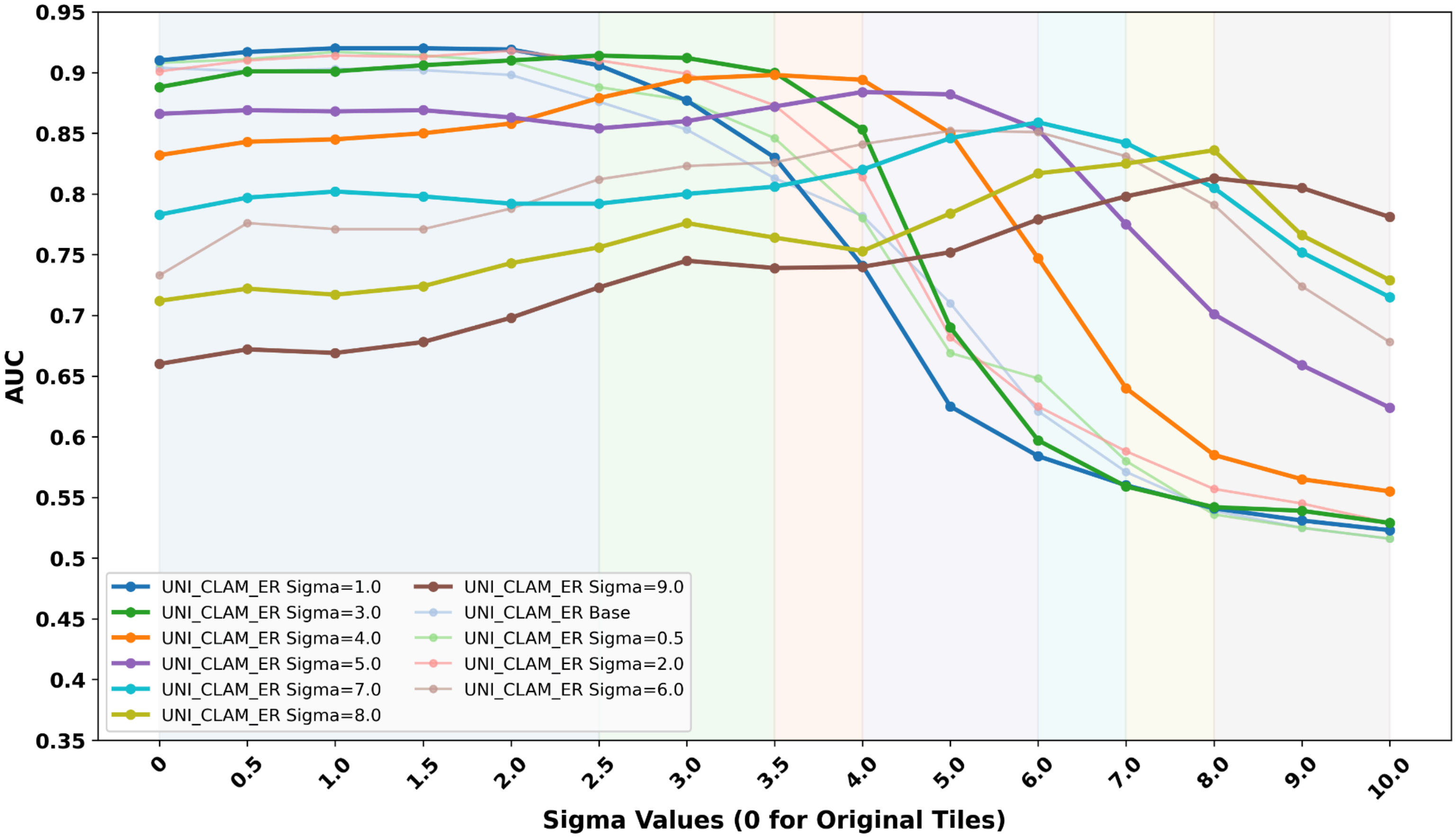

Figure S9. AUC performance of one baseline and ten blur-resistant UNI_CLAM_ER models and cut-offs for model selection. AUC of one baseline and the ten blur-resistant UNI_CLAM_ER models evaluated on validation sets with increasing levels of Gaussian blur (σ ranging from 0 to 10). Each UNI_CLAM_ER model was trained on features extracted via UNI using tiles blurred with a fixed sigma level for ER positive and

negative status classification. The x-axis represents the sigma values applied to the validation tiles, simulating an increasing level of blur. The y-axis shows the AUC of each model's prediction at the slide level. Colored background regions denote the empirically defined blur category cut-offs used in MoE strategy for model selection.

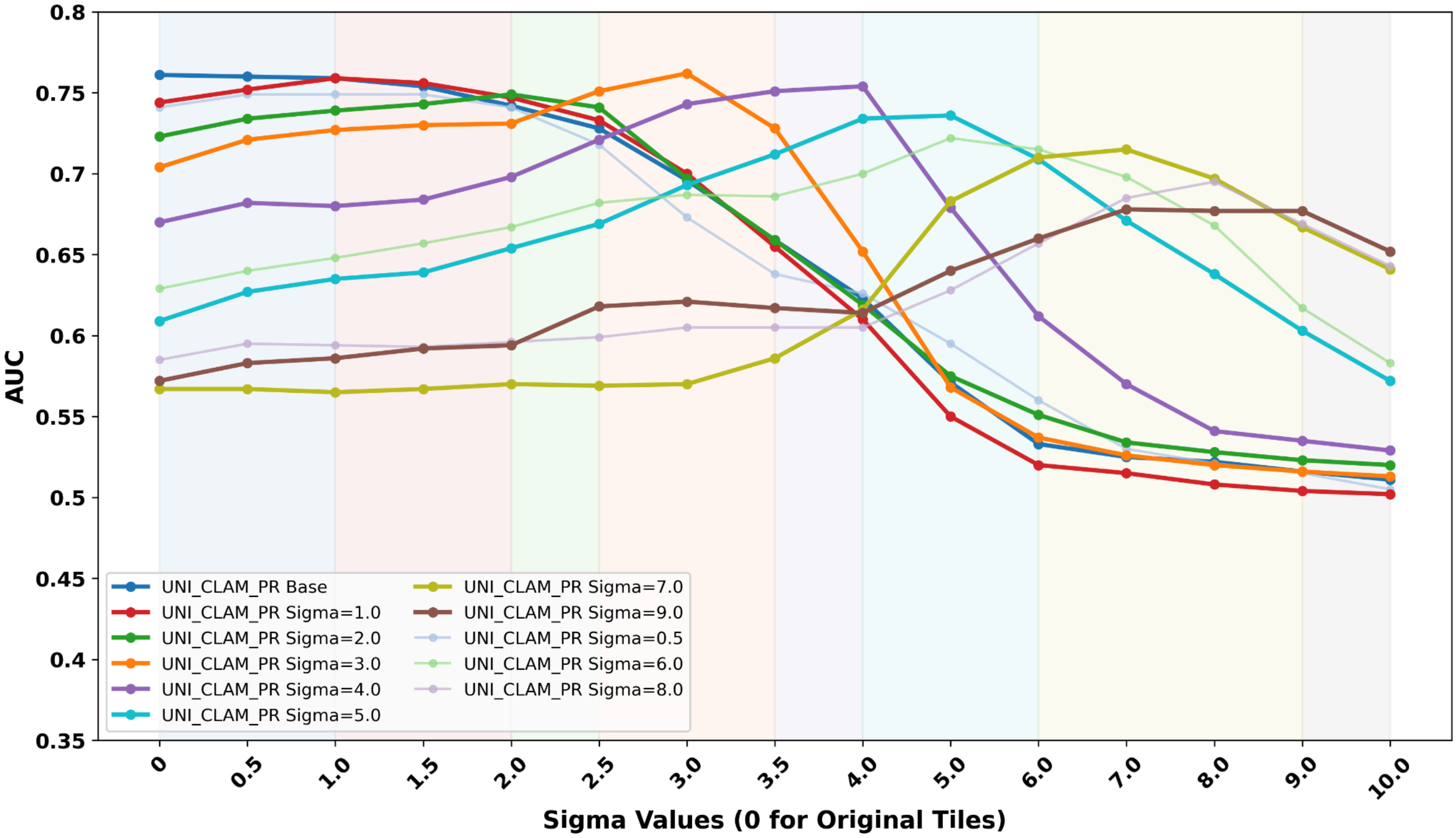

Figure S10. AUC performance of one baseline and ten blur-resistant UNI_CLAM_PR models and cut-offs for model selection. AUC of one baseline and the ten blur-resistant UNI_CLAM_PR models evaluated on validation sets with increasing levels of Gaussian blur (σ ranging from 0 to 10). Each UNI_CLAM_PR model was trained on features extracted via UNI using tiles blurred with a fixed sigma level for PR positive and negative status classification. The x-axis represents the sigma values applied to the validation tiles, simulating an increasing level of blur. The y-axis shows the AUC of each model's prediction at the slide level. Colored background regions denote the empirically defined blur category cut-offs used in MoE strategy for model selection.

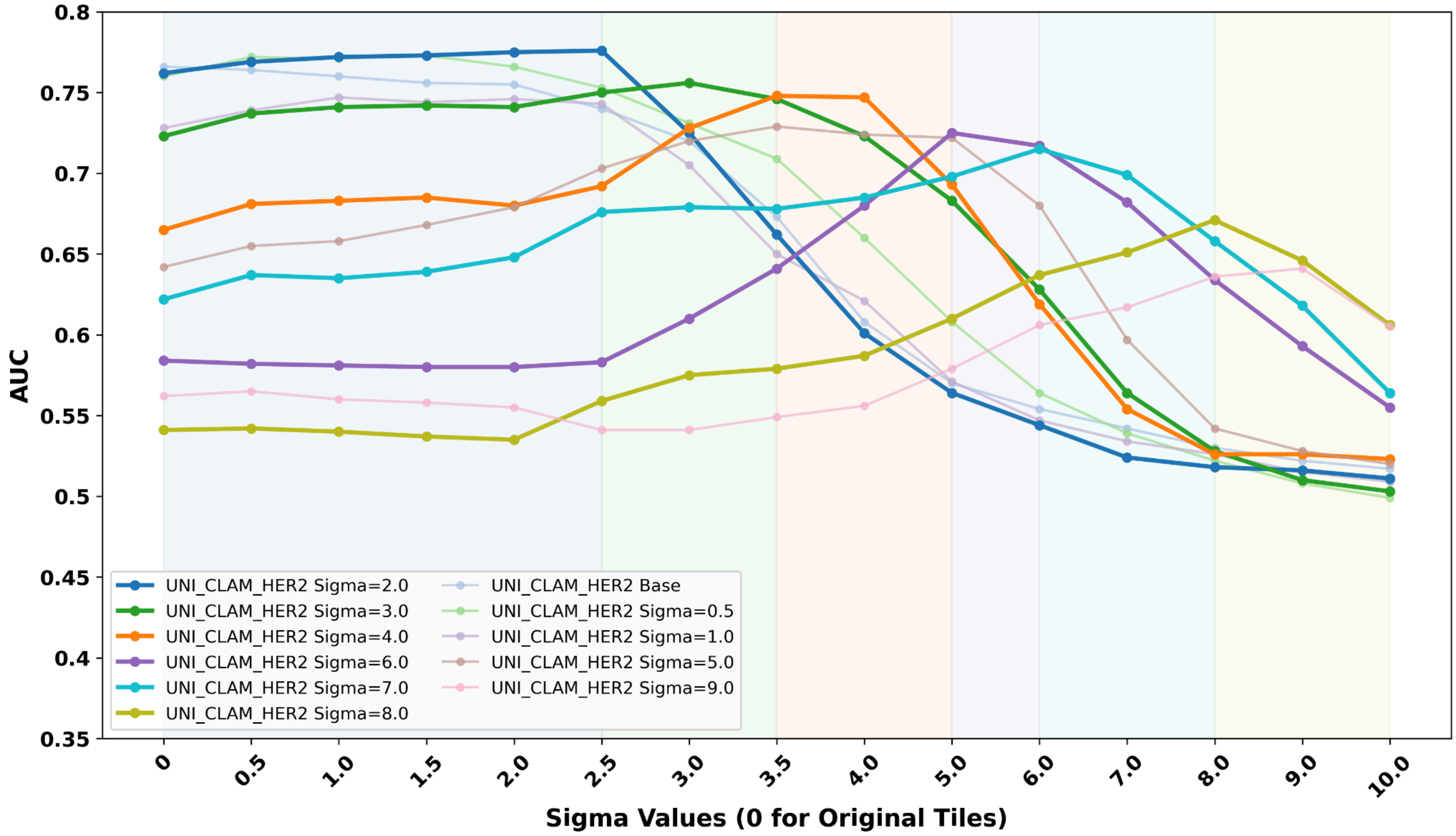

Figure S11. AUC performance of one baseline and ten blur-resistant UNI_CLAM_Her2 models and cut-offs for model selection. AUC of one baseline and the ten blur-resistant UNI_CLAM_Her2 models evaluated on validation sets with increasing levels of Gaussian blur (σ ranging from 0 to 10). Each UNI_CLAM_Her2 model was trained on features extracted via UNI using tiles blurred with a fixed sigma level for Her2 positive and negative status classification. The x-axis represents the sigma values applied to the validation tiles, simulating an increasing level of blur. The y-axis shows the AUC of each model's prediction at the slide level. Colored background regions denote the empirically defined blur category cut-offs used in MoE strategy for model selection.

Table S6. Model selection for MoE experts by sigma range and Laplacian Variance thresholds

MoE Models	Sigma Ranges	LV Ranges	Expert Models
MoE-CNN_simple	[0, 1.5)	36.13 (mean of median LV values for σ = 1.5 and 2.0) < LV	Model_Base
	[1.5, 2.5)	7.54 (mean of median LV values for σ = 2.5 and 3.0) < LV ≤ 36.13	Model_0.5
	[2.5, 3.5)	3.76 (mean of median LV values for σ = 3.5 and 4.0) < LV ≤ 7.54	Model_3.0
	[3.5, 5.0)	2.52 (mean of median LV values for σ = 5.0 and 6.0) < LV ≤ 3.76	Model_4.0
	[5.0, 10.0]	LV ≤ 2.52	Model_5.0
MoE-CNN_CLAM	[0, 1.5)	36.13 (mean of median LV values for σ = 1.5 and 2.0) < LV	CNN_CLAM_Base
	[1.5, 2.5)	7.54 (mean of median LV values for σ = 2.5 and 3.0) < LV ≤ 36.13	CNN_CLAM_2.0
	[2.5, 3.5)	3.76 (mean of median LV values for σ = 3.5 and 4.0) < LV ≤ 7.54	CNN_CLAM_3.0
	[3.5, 5.0)	2.52 (mean of median LV values for σ = 5.0 and 6.0) < LV ≤ 3.76	CNN_CLAM_4.0
	[5.0, 7.0)	2.13 (mean of median LV values for σ = 7.0 and 8.0) < LV ≤ 2.52	CNN_CLAM_5.0
	[7.0, 9.0)	1.93 (mean of median LV values for σ = 9.0 and 10.0) < LV ≤ 2.13	CNN_CLAM_8.0
	[9.0, 10.0]	LV ≤ 1.93	CNN_CLAM_9.0
MoE-UNI_CLAM	[0, 3.0)	4.96 (mean of median LV values for σ = 3.0 and 3.5) < LV	UNI_CLAM_1.0
	[3.0, 3.5)	3.76 (mean of median LV values for σ = 3.5 and 4.0) < LV ≤ 4.96	UNI_CLAM_3.0
	[3.5, 5.0)	2.52 (mean of median LV values for σ = 5.0 and 6.0) < LV ≤ 3.76	UNI_CLAM_4.0
	[5.0, 6.0)	2.29 (mean of median LV values for σ = 6.0 and 7.0) < LV ≤ 2.52	UNI_CLAM_5.0
	[6.0, 8.0)	2.02 (mean of median LV values for σ = 8.0 and 9.0) < LV ≤ 2.29	UNI_CLAM_7.0
	[8.0, 10.0]	LV ≤ 2.02	UNI_CLAM_9.0
MoE-UNI_CLAM_ER	[0, 2.5)	7.54 (mean of median LV values for σ = 2.5 and 3.0) < LV	UNI_CLAM_ER_1.0
	[2.5, 3.5)	3.76 (mean of median LV values for σ = 3.5 and 4.0) < LV ≤ 7.54	UNI_CLAM_ER_3.0
	[3.5, 4.0)	3.00 (mean of median LV values for σ = 4.0 and 5.0) < LV ≤ 3.76	UNI_CLAM_ER_4.0
	[4.0, 6.0)	2.29 (mean of median LV values for σ = 6.0 and 7.0) < LV ≤ 3.00	UNI_CLAM_ER_5.0
	[6.0, 7.0)	2.13 (mean of median LV values for σ = 7.0 and 8.0) < LV ≤ 2.29	UNI_CLAM_ER_7.0
	[7.0, 8.0)	2.02 (mean of median LV values for σ = 8.0 and 9.0) < LV ≤ 2.13	UNI_CLAM_ER_8.0
	[8.0, 10.0]	LV ≤ 2.02	UNI_CLAM_ER_9.0
MoE-UNI_CLAM_PR	[0, 1.0)	125.93 (mean of median LV values for σ = 1.0 and 1.5) < LV	UNI_CLAM_PR_Base
	[1.0, 2.0)	14.47 (mean of median LV values for σ = 2.0 and 2.5) < LV ≤ 125.93	UNI_CLAM_PR_1.0
	[2.0, 2.5)	7.54 (mean of median LV values for σ = 2.5 and 3.0) < LV ≤ 14.47	UNI_CLAM_PR_2.0
	[2.5, 3.5)	3.76 (mean of median LV values for σ = 3.5 and 4.0) < LV ≤ 7.54	UNI_CLAM_PR_3.0
	[3.5, 4.0)	3.00 (mean of median LV values for σ = 4.0 and 5.0) < LV ≤ 3.76	UNI_CLAM_PR_4.0
	[4.0, 6.0)	2.29 (mean of median LV values for σ = 6.0 and 7.0) < LV ≤ 3.00	UNI_CLAM_PR_5.0
	[6.0, 9.0)	1.93 (mean of median LV values for σ = 9.0 and 10.0) < LV ≤ 2.29	UNI_CLAM_PR_7.0
	[9.0, 10.0]	LV ≤ 1.93	UNI_CLAM_PR_9.0
MoE-UNI_CLAM_Her2	[0, 2.5)	7.54 (mean of median LV values for σ = 2.5 and 3.0) < LV	UNI_CLAM_Her2_2.0
	[2.5, 3.5)	3.76 (mean of median LV values for σ = 3.5 and 4.0) < LV ≤ 7.54	UNI_CLAM_Her2_3.0
	[3.5, 5.0)	2.52 (mean of median LV values for σ = 5.0 and 6.0) < LV ≤ 3.76	UNI_CLAM_Her2_4.0
	[5.0, 6.0)	2.29 (mean of median LV values for σ = 6.0 and 7.0) < LV ≤ 2.52	UNI_CLAM_Her2_6.0
	[6.0, 8.0)	2.02 (mean of median LV values for σ = 8.0 and 9.0) < LV ≤ 2.29	UNI_CLAM_Her2_7.0
	[8.0, 10.0]	LV ≤ 2.02	UNI_CLAM_Her2_8.0

Table S7. AUC comparison between baseline and MoE models using 200 WSIs with the highest level of blur in a real-world WSI dataset

Tasks	Baseline (UNI_CLAM)	MoE (MoE-UNI_CLAM)
Grade	0.930	0.947
ER	0.915	0.915
Her2	0.791	0.806
PR	0.775	0.785
